# The perfect waveguide coupler with universal impedance matching and transformation optics

**DOI:** 10.1515/nanoph-2023-0771

**Published:** 2024-02-22

**Authors:** Myeongjin Kim, Q-Han Park

**Affiliations:** Department of Physics, 34973 Korea University, Seoul, 02841, Republic of Korea

**Keywords:** universal impedance matching, transformation optics, waveguide coupler, fiber-to-chip coupling, optical interconnects

## Abstract

Efficient energy transfer is crucial in electromagnetic communication. Therefore, producing a waveguide coupler that achieves broadband, nonreflective transmission is a challenging task. With the advancement of silicon-based integrated photonic circuits, fiber-to-chip coupling has become increasingly important. Although various couplers have been developed for fiber-to-chip coupling, they often have limitations such as long coupling length, low coupling efficiency, and narrow bandwidth. This is due to the inability to eliminate reflections between the two waveguides. Here, we introduce a method using universal impedance matching theory and transformation optics to eliminate reflections between two waveguides. The coupler, called the universal impedance matching coupler, using this method has the shortest subwavelength coupling length, a 99.9 % coupling efficiency, and a broad bandwidth.

## 1 Introduction

When electromagnetic waves traverse between two waveguides with distinct cross sections and internal filling materials, reflection occurs as a result of disparities in mode shape and impedance. Diverse coupler designs have been introduced to enhance coupling efficiency by addressing mode shape and impedance mismatches. Notably, Klopfenstein’s tapered coupler, based on transmission-line theory, shows a significant advancement despite the drawback of requiring an extended tapering length [1]. In the context of fiber-to-chip coupling, two categories of couplers, namely Edge Couplers and Grating Couplers, have emerged, each with its specific limitations [2], [3]. Edge Couplers require a significant size, featuring a taper length exceeding 20 μm and a taper angle of 25° or less based on the mode field diameter [3], [4], [5], [6], [7], [8], [9], [10]. In contrast, Grating Couplers exhibit a limited bandwidth and lower coupling efficiency, often at 90 % or less [11], [12], [13], [14], [15]. These limitations stem from challenges in effectively eliminating reflections between the two waveguides. Despite efforts to employ new approaches such as transformation optics [16], the problem of coupling loss due to reflection caused by impedance mismatch at the interface remains unsolved [17], [18], [19], [20]. To date, no systematic approach has been devised to independently address both mismatches, shape and impedance, in diverse coupling scenarios between distinct waveguides.

In this study, we introduce an ideal coupler that effectively eliminates both mode shape and impedance disparities, enabling broadband, nonreflective transmission. Utilizing transformation optics, we replace waveguides of various sizes filled with isotropic mediums with a single-size waveguide filled with an anisotropic medium. The mode of this single-size PEC waveguide is primarily determined by its cross-sectional boundary, effectively eliminating mode shape mismatches through this conversion. Additionally, impedance mismatches can be resolved by employing the Universal Impedance Matching (UIM) theory [21], which tackles inverse scattering problems using admittance and impedance functions. We design an UIM coupler by inversely transforming parameters obtained through the inverse scattering process.

To validate our approach, we conducted a comprehensive 3D numerical simulation confirming that the UIM coupler consistently achieves over 99.9 % transmission, irrespective of various coupling conditions such as frequency, modes, tapering length, and cross-sectional shape. Notably, the UIM coupler exhibits spatially and temporally dispersive anisotropic permittivity and permeability tensors.

## 2 Design and performance of UIM coupler

### 2.1 Theoretical background

Our ultimate goal is to perfectly couple two waveguides with different cross sections and internal materials, and we introduce a theory that will make this possible. While the theory can be applied to waveguides of various shapes, we will concentrate on a rectangular waveguide for the sake of clarity. A basic rectangular waveguide has dimensions *a* × *b*, enclosed by a Perfect Electric Conductor (PEC) boundary and filled with an arbitrary material. When a monochromatic electromagnetic (EM) wave with angular frequency *ω* travels along the *z* axis, the PEC boundary condition establishes the shape of EM wave mode profiles within the waveguide. These profiles, identified by non-negative integer mode number *n* and *m* of wavenumbers $k_{x}=\frac{n\pi }{a},k_{y}=\frac{m\pi }{b}$ , are expressed as follows:

(1) $$\begin{array}{c} E_{x}=E_{x,nm}\left(z\right)\cos\left(k_{x}x\right)\sin\left(k_{y}y\right)\\ E_{y}=E_{y,nm}\left(z\right)\sin\left(k_{x}x\right)\cos\left(k_{y}y\right)\\ E_{z}=E_{z,nm}\left(z\right)\sin\left(k_{x}x\right)\sin\left(k_{y}y\right)\\ H_{x}=H_{x,nm}\left(z\right)\sin\left(k_{x}x\right)\cos\left(k_{y}y\right)\\ H_{y}=H_{y,nm}\left(z\right)\cos\left(k_{x}x\right)\sin\left(k_{y}y\right)\\ H_{z}=H_{z,nm}\left(z\right)\cos\left(k_{x}x\right)\cos\left(k_{y}y\right) \end{array}$$

Then, the Maxwell equation incorporating the time-dependent factor *e* ^−*iωt*^ of monochromatic wave reduces to

(2) $$\begin{array}{c} \partial_{z}H_{y,nm}\left(z\right)+k_{y}H_{z,nm}\left(z\right)=i\omega \varepsilon_{x}E_{x,nm}\left(z\right)\;\left(a\right)\\ \partial_{z}H_{x,nm}\left(z\right)+k_{x}H_{z,nm}\left(z\right)=-i\omega \varepsilon_{y}E_{y,nm}\left(z\right)\;\left(b\right)\\ k_{x}H_{y,nm}\left(z\right)-k_{y}H_{x,nm}\left(z\right)=i\omega \varepsilon_{z}E_{z,nm}\left(z\right)\;\left(c\right)\\ \partial_{z}E_{y,nm}\left(z\right)-k_{y}E_{z,nm}\left(z\right)=-i\omega \mu_{x}H_{x,nm}\left(z\right)\;\left(d\right)\\ \partial_{z}E_{x,nm}\left(z\right)-k_{x}E_{z,nm}\left(z\right)=i\omega \mu_{y}H_{y,nm}\left(z\right)\;\left(e\right)\\ k_{x}E_{y,nm}\left(z\right)-k_{y}E_{x,nm}\left(z\right)=i\omega \mu_{z}H_{z,nm}\left(z\right)\;\left(f\right) \end{array}$$

**Figure 1:**
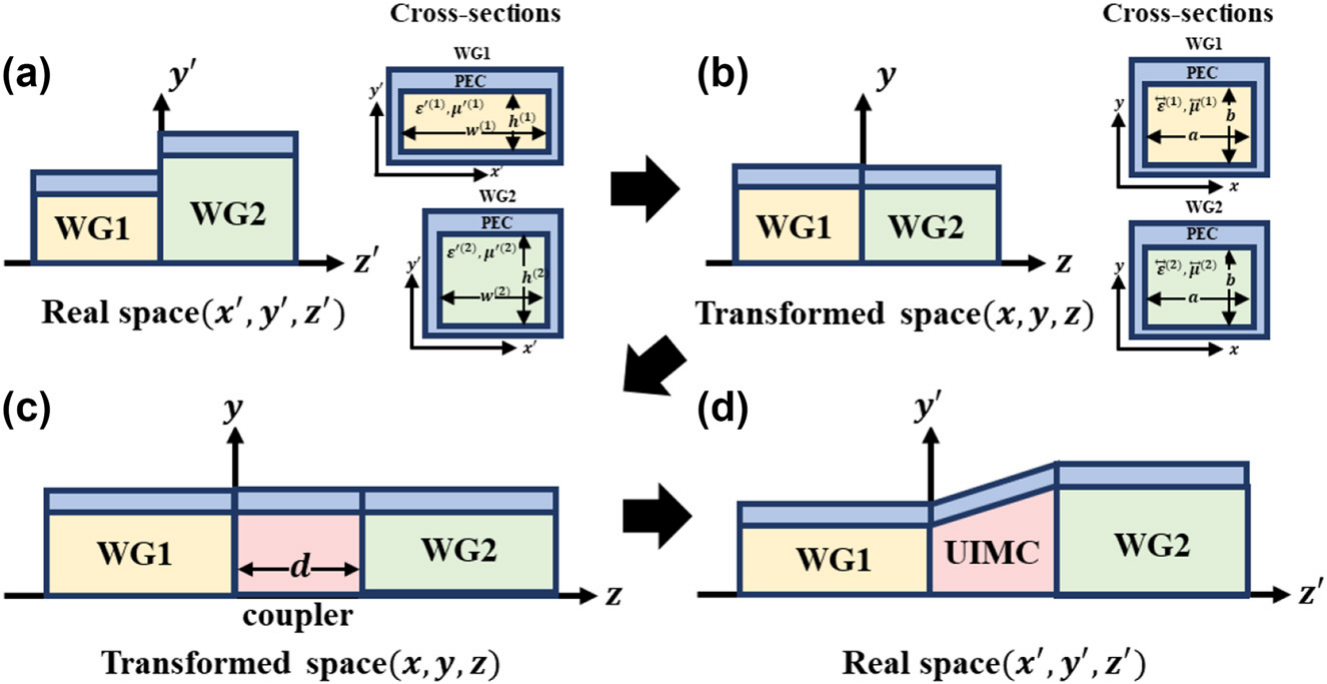
Schematic of coupling (a) two different waveguides named waveguide 1 (WG1) and waveguide 2 (WG2) (left), with different corresponding cross sections (right). (b) Through transformation, the cross sections of WG1 and WG2 become the same (left), and the corresponding cross sections (right). (c) Insert coupler with the same cross section and thickness *d*. (d) Implementation of perfect waveguide coupler (UIMC) through inverse transformation.

As depicted in Figure 1, we examine the connection of two waveguides, denoted as WG1 and WG2, each having distinct cross sections characterized by widths represented as $w^{\left(j\right)}$ and heights as $h^{\left(j\right)}$ . These waveguides are filled with an isotropic medium featuring permittivity $\varepsilon^{\prime \left(j\right)}$ and permeability $\mu^{\prime \left(j\right)}\left(j=1,2\right)$ . When $w^{\left(1\right)}\neq w^{\left(2\right)}$ or $h^{\left(1\right)}\neq h^{\left(2\right)}$ , the modes of WG1 and WG2 do not align, resulting in the coupling between modes with different mode numbers. This complicates the analysis of propagation dynamics and the design of the coupler. To mitigate the complexity arising from the mismatch in mode shapes, we propose a straightforward method based on transformation optics. This method aims to standardize the cross sections of WG1 and WG2 to a common reference cross section with dimensions *a* × *b*. For this purpose, we introduce a transformed space $\left(x^{\left(j\right)},y^{\left(j\right)},z^{\left(j\right)}\right)$ in contrast to the real space $\left(x^{\prime },y^{\prime },z^{\prime }\right)$ as in Figure 1-(a),

(3) $$x=\frac{a}{w^{\left(j\right)}}x^{\prime },y=\frac{b}{h^{\left(j\right)}}y^{\prime },z=z^{\prime }\;\left(j=1,2\right)$$

This transformation aligns WG1 and WG2 to possess the same cross section and mode shape, as shown in Figure 1-(b). Consequently, mode coupling exclusively occurs between mode of identical mode numbers, owing to the orthogonality of these modes [22]. Following the coordinate transformation, as outlined in transformation optics [23], the permittivity and permeability undergo a corresponding transformation. Specifically, the originally isotropic medium denoted by $\left(\varepsilon^{\prime \left(j\right)},\mu^{\prime \left(j\right)}\right)$ within WG1 and WG2 in real space is converted into a specific anisotropic medium $\left({\overset{↔}{\varepsilon }}^{\left(j\right)},{\overset{↔}{\mu }}^{\left(j\right)}\right)$ in transformed space as follows:

(4) $${\overset{↔}{\varepsilon }}^{\left(j\right)}=\frac{1}{\det\left(\Lambda^{\left(j\right)}\right)}\Lambda^{\left(j\right)}\left(\begin{array}{ccc} \varepsilon^{\prime \left(j\right)} & 0 & 0\\ 0 & {\varepsilon^{\prime }}^{\left(j\right)} & 0\\ 0 & 0 & \varepsilon^{\prime \left(j\right)} \end{array}\right)\Lambda^{\left(j\right)T}$$

and similar expression for the permeability ${\overset{↔}{\mu }}^{\left(j\right)}$ with the positions of *ɛ* and *μ* interchanged. The Jacobian matrix Λ governing the change of coordinate basis is

(5) $$\Lambda^{\prime }=\left(\begin{array}{ccc} \frac{\partial x}{\partial x^{\prime }} & \frac{\partial x}{\partial y^{\prime }} & \frac{\partial x}{\partial z^{\prime }}\\ \frac{\partial y}{\partial x^{\prime }} & \frac{\partial y}{\partial y^{\prime }} & \frac{\partial y}{\partial z^{\prime }}\\ \frac{\partial z}{\partial x^{\prime }} & \frac{\partial z}{\partial y^{\prime }} & \frac{\partial z}{\partial z^{\prime }} \end{array}\right)=\left(\begin{array}{ccc} \frac{a}{w^{\left(j\right)}} & 0 & 0\\ 0 & \frac{b}{h^{\left(j\right)}} & 0\\ 0 & 0 & 1 \end{array}\right)\;$$

In component, they are given by

(6) $$\begin{array}{c} {\overset{↔}{\varepsilon }}^{\left(j\right)}=\mathrm{diag}\left(\varepsilon_{x}^{\left(j\right)},\varepsilon_{y}^{\left(j\right)},\varepsilon_{z}^{\left(j\right)}\right),N^{\left(j\right)}\equiv \frac{ah^{\left(j\right)}}{bw^{\left(j\right)}}\text{,}\left(j=1,2\right)\\ \varepsilon_{x}^{\left(j\right)}=N^{\left(j\right)}\varepsilon^{\prime \left(j\right)}\text{ , }\varepsilon_{y}^{\left(j\right)}=\frac{\varepsilon^{\prime \left(j\right)}}{N^{\left(j\right)}}\text{ , }\varepsilon_{z}^{\left(j\right)}=\frac{w^{\left(j\right)}h^{\left(j\right)}}{ab}\varepsilon^{\prime \left(j\right)}\\ \mu_{x}^{\left(j\right)}=N^{\left(j\right)}\mu^{\prime \left(j\right)}\text{ , }\mu_{y}^{\left(j\right)}=\frac{\mu^{\prime \left(j\right)}}{N^{\left(j\right)}}\text{ , }\mu_{z}^{\left(j\right)}=\frac{w^{\left(j\right)}h^{\left(j\right)}}{ab}\mu^{\prime \left(j\right)} \end{array}$$

In the transformed space, the reflection between WG1 and WG2, which now possess the same cross section, is solely determined by the impedance mismatch arising from the differing internal materials $\left({\overset{↔}{\varepsilon }}^{\left(1\right)}\neq {\overset{↔}{\varepsilon }}^{\left(2\right)},{\overset{↔}{\mu }}^{\left(1\right)}\neq {\overset{↔}{\mu }}^{\left(2\right)}\right)$ . To eliminate the impedance mismatch between WG1 and WG2, a coupler with the same cross section and thickness d is inserted between the two, as depicted in Figure 1-(c).

It is important to emphasize that the coupler must bridge the gap between WG1 and WG2, which initially had different cross sections prior to the transformation. To ensure that the transformed coupler maintains the same cross section, we require a transformation that is dependent on *z*. Additionally, the permittivity and permeability of the material within the coupler should be also functions of *z* and exhibit the same type of anisotropy induced by the transformation in order to maintain impedance matching consistently. Explicitly with an arbitrary dimensionless constant *N*, these properties are characterized by

(7) $$\begin{array}{c} \varepsilon_{x}\equiv N\varepsilon_{r}\left(z\right),\varepsilon_{y}\equiv \frac{\varepsilon_{r}\left(z\right)}{N},\varepsilon_{z}\equiv \varepsilon_{z}\left(z\right)\\ \mu_{x}\equiv N\mu_{r}\left(z\right),\mu_{y}\equiv \frac{\mu_{r}\left(z\right)}{N},\mu_{z}\equiv \mu_{z}\left(z\right) \end{array}$$

Let’s first address the problem of impedance matching. If we confine our analysis to the transverse magnetic (TM) mode $\left(H_{z}=0\right)$ , the reduced Maxwell equation (2-f) and the divergence free condition ∇ ⋅ *B* = 0 can be solved in terms of two potential functions $Y\left(z\right),\Phi^{E}\left(z\right)$ [21] to yield,

(8) $$\begin{array}{c} H_{x,nm}\left(z\right)=k_{y}\left(\frac{Y}{N}\right)\Phi^{E},H_{y,nm}\left(z\right)=-k_{x}\left(NY\right)\Phi^{E}\\ E_{x,nm}\left(z\right)=-k_{x}\Phi^{E},E_{y,nm}\left(z\right)=-k_{y}\Phi^{E}\\ E_{z,nm}\left(z\right)=-\left[\left(Nk_{x}^{2}+k_{y}^{2}/N\right)/i\omega \varepsilon_{z}\right]Y\Phi^{E} \end{array}$$

The remaining Maxwell equation consistently reduces to

(9) $$\begin{array}{c} \partial_{z}Y+\left[i\omega \mu_{r}-\frac{i}{\omega \varepsilon_{z}}\left(Nk_{x}^{2}+\frac{k_{y}^{2}}{N}\right)\right]Y^{2}=i\omega \varepsilon_{r}\\ \partial_{z}\left(Y\Phi^{E}\right)=i\omega \varepsilon_{r}\Phi^{E}\cdot \end{array}$$

Similarly, if we confine our analysis to the transverse electric (TE) mode $\left(E_{z}=0\right)$ , the reduced Maxwell equation (2-c) and the divergence free condition ∇·*D* = 0 can be solved in terms of two potential functions $Z\left(z\right),\Phi^{H}\left(z\right)$ to yield,

(10) $$\begin{array}{c} E_{x,nm}\left(z\right)=k_{y}\left(\frac{Z}{N}\right)\Phi^{H},E_{y,nm}\left(z\right)=-k_{x}\left(NZ\right)\Phi^{H}\\ H_{x,nm}\left(z\right)=k_{x}\Phi^{H},H_{y,nm}\left(z\right)=k_{y}\Phi^{H}\\ H_{z,nm}\left(z\right)=-\left[\left(Nk_{x}^{2}+k_{y}^{2}/N\right)/i\omega \mu_{z}\right]Z\Phi^{H} \end{array}$$

together with the remaining equation

(11) $$\begin{array}{c} \partial_{z}Z+\left[i\omega \varepsilon_{r}-\frac{i}{\omega \mu_{z}}\left(Nk_{x}^{2}+\frac{k_{y}^{2}}{N}\right)\right]Z_{x}^{2}=i\omega \mu_{r}\\ \partial_{z}\left(Z\Phi^{H}\right)=i\omega \mu_{r}\Phi^{H}\cdot \end{array}$$

Simultaneously, we can evaluate the admittance and impedance of the (*n*, *m*)-TM and TE modes in WG1 and WG2. They are determined by

(12) $$\begin{array}{c} Y^{\left(j\right)}\equiv \frac{\omega \varepsilon^{\prime \left(j\right)}}{k_{z}^{TM\left(j\right)}},Z^{\left(j\right)}\equiv \frac{\omega \mu^{\prime \left(j\right)}}{k_{z}^{TE\left(j\right)}}\text{,}\;\left(j=1,2\right)\\ k_{z}^{TM\left(j\right)}={\left[\omega^{2}{\varepsilon^{\prime }}^{\left(j\right)}{\mu^{\prime }}^{\left(j\right)}-\frac{{\varepsilon^{\prime }}^{\left(j\right)}}{\varepsilon_{z}^{\left(j\right)}}\left(N^{\left(j\right)}k_{x}^{2}+\frac{k_{y}^{2}}{N^{\left(j\right)}}\right)\right]}^{\frac{1}{2}}\\ k_{z}^{TE\left(j\right)}={\left[\omega^{2}{\varepsilon^{\prime }}^{\left(j\right)}{\mu^{\prime }}^{\left(j\right)}-\frac{{\mu^{\prime }}^{\left(j\right)}}{\mu_{z}^{\left(j\right)}}\left(N^{\left(j\right)}k_{x}^{2}+\frac{k_{y}^{2}}{N^{\left(j\right)}}\right)\right]}^{\frac{1}{2}} \end{array}$$

On the other hand, the admittance and impedance of the coupler are functions of *z* satisfying equations (9) and (11), respectively. Their boundary conditions at both *z* = 0 and *z* = *d* can be chosen to match the constant admittance and impedance of WG1 and WG2. Explicitly, the matching boundary conditions are,

(13) $$Y\left(0\right)=Y^{\left(1\right)},\;Y\left(d\right)=Y^{\left(2\right)},\;\text{Z}\left(0\right)=Z^{\left(1\right)},\;\text{Z}\left(d\right)=Z^{\left(2\right)}\text{,}$$

A particular solution satisfying the boundary condition and yielding real and constant permittivity and permeability can be readily found with the result,

(14) $$\begin{array}{c} Y\left(z\right)=\frac{1}{N}\left[\frac{\begin{array}{c} 2{\bar{Y}}^{\left(1\right)}{\bar{Y}}^{\left(2\right)}+i\sqrt{{\bar{Y}}^{\left(1\right)}{\bar{Y}}^{\left(2\right)}}\\ \left({\bar{Y}}^{\left(2\right)}-{\bar{Y}}^{\left(1\right)}\right)\sin\left(\frac{\pi }{d}z\right) \end{array}}{\left({\bar{Y}}^{\left(2\right)}+{\bar{Y}}^{\left(1\right)}\right)+\left({\bar{Y}}^{\left(2\right)}-{\bar{Y}}^{\left(1\right)}\right)\cos\left(\frac{\pi }{d}z\right)}\right]\\ Z\left(z\right)=N\left[\frac{\begin{array}{c} 2{\bar{Z}}^{\left(1\right)}{\bar{Z}}^{\left(2\right)}+i\sqrt{{\bar{Z}}^{\left(1\right)}{\bar{Z}}^{\left(2\right)}}\\ \left({\bar{Z}}^{\left(2\right)}-{\bar{Z}}^{\left(1\right)}\right)\sin\left(\frac{\pi }{d}z\right) \end{array}}{\left({\bar{Z}}^{\left(2\right)}+{\bar{Z}}^{\left(1\right)}\right)+\left({\bar{Z}}^{\left(2\right)}-{\bar{Z}}^{\left(1\right)}\right)\cos\left(\frac{\pi }{d}z\right)}\right]\\ {\bar{Y}}^{\left(j\right)}=N^{\left(j\right)}Y^{\left(j\right)},\;{\bar{Z}}^{\left(j\right)}=\frac{Z^{\left(j\right)}}{N^{\left(j\right)}},\;N=\sqrt{N^{\left(1\right)}N^{\left(2\right)}} \end{array}$$

We observe that the permittivity and permeability can be directly obtained from equations (9) and (11) by handling the real and imaginary parts of the equations separately [21] and utilizing the outcomes presented in (14). This serves as an example of an explicit solution to the inverse scattering problem. The results are provided as follows:

(15) $$\begin{array}{c} \varepsilon_{r}=\frac{\pi }{2d\omega }\sqrt{Y^{\left(1\right)}Y^{\left(2\right)}},\;\mu_{r}=\frac{\pi }{2d\omega }\sqrt{Z^{\left(1\right)}Z^{\left(2\right)}},\\ \varepsilon_{z}=\frac{2d}{\omega \pi }\left(Nk_{x}^{2}+\frac{k_{y}^{2}}{N}\right){\left(\sqrt{Z^{\left(1\right)}Z^{\left(2\right)}}-\frac{1}{\sqrt{Y^{\left(1\right)}Y^{\left(2\right)}}}\right)}^{-1}\\ \mu_{z}=\frac{2d}{\omega \pi }\left(Nk_{x}^{2}+\frac{k_{y}^{2}}{N}\right){\left(\sqrt{Y^{\left(1\right)}Y^{\left(2\right)}}-\frac{1}{\sqrt{Z^{\left(1\right)}Z^{\left(2\right)}}}\right)}^{-1} \end{array}$$

The parameters specified in (15), termed the Universal Impedance Matching (UIM) parameter, facilitate perfect impedance matching between two waveguides, WG1 and WG2, entirely eliminating reflection.

Up to this point, our work has primarily involved the Universal Impedance Matching (UIM) in transformed space. However, in real space, the coupler and UIM parameters need to be reverted through the inverse transformation, as depicted in Figure 1-(d). In the cases of WG1 and WG2, they return to their original shapes as shown in Figure 1-(a) by inversely applying the transformation equation (2). Since the original cross sections of WG1 and WG2 differ, the UIM coupler bridging these waveguides must be tapered to ensure the continuity of the coordinate transformation at the interfaces between the waveguide and coupler. If this transformation continuity is not maintained, reflection can occur at the interface between the waveguide and the coupler [24]. Therefore, we employ an inverse transformation that linearly interpolates between two parts as follows:

(16) $$\begin{array}{c} x^{\prime }=\frac{1}{ad}\left[w^{\left(1\right)}d+\left(w^{\left(2\right)}-w^{\left(1\right)}\right)z\right]x,\\ y^{\prime }=\frac{1}{bd}\left[h^{\left(1\right)}d+\left(h^{\left(2\right)}-h^{\left(1\right)}\right)z\right]y,\;z^{\prime }=z \end{array}$$

The Jacobian transformation matrix is defined as

(17) $$\begin{array}{ll} \Lambda & =\left(\begin{array}{ccc} \frac{\partial x^{\prime }}{\partial x} & \frac{\partial x^{\prime }}{\partial y} & \frac{\partial x^{\prime }}{\partial z}\\ \frac{\partial y^{\prime }}{\partial x} & \frac{\partial y^{\prime }}{\partial y} & \frac{\partial y^{\prime }}{\partial z}\\ \frac{\partial z^{\prime }}{\partial x} & \frac{\partial z^{\prime }}{\partial y} & \frac{\partial z^{\prime }}{\partial z} \end{array}\right)\\ & =\left(\begin{array}{ccc} R_{x}\left(z^{\prime }\right) & 0 & \tan\alpha \left(x^{\prime },z^{\prime }\right)\\ 0 & R_{y}\left(z^{\prime }\right) & \tan\beta \left(y^{\prime },z^{\prime }\right)\\ 0 & 0 & 1 \end{array}\right) \end{array}$$

Then, the UIM parameters in real space are given by

(18) $$\begin{array}{ll} {\overset{↔}{\varepsilon^{\prime }}}^{\mathrm{UIMT}} & =\frac{1}{\det\left(\Lambda \right)}\Lambda \left(\begin{array}{ccc} \varepsilon_{x} & 0 & 0\\ 0 & \varepsilon_{y} & 0\\ 0 & 0 & \varepsilon_{z} \end{array}\right)\Lambda^{\text{T}}\\ & =\frac{1}{R_{x}R_{y}}\left(\begin{array}{ccc} \varepsilon_{x}R_{x}^{2}+\varepsilon_{z}\tan^{2}\alpha & \varepsilon_{z}\tan\alpha \tan\beta & \varepsilon_{z}\tan\alpha \\ \varepsilon_{z}\tan\alpha \tan\beta & \varepsilon_{y}R_{y}^{2}+\varepsilon_{z}\tan^{2}\beta & \varepsilon_{z}\tan\beta \\ \varepsilon_{z}\tan\alpha & \varepsilon_{z}\tan\beta & \varepsilon_{z} \end{array}\right) \end{array}$$

and similar expression for the permeability ${\overset{↔}{\mu^{\prime }}}^{\mathrm{UIMT}}$ with the positions of *ɛ* and *μ* interchanged. Here,

(19) $$\begin{array}{c} R_{x}=\frac{x^{\prime }}{x}=\frac{1}{ad}\left[w^{\left(1\right)}d+\left(w^{\left(2\right)}-w^{\left(1\right)}\right)z^{\prime }\right],\\ R_{y}=\frac{y^{\prime }}{y}=\frac{1}{bd}\left[h^{\left(1\right)}d+\left(h^{\left(2\right)}-h^{\left(1\right)}\right)z^{\prime }\right],\\ \tan\alpha ==\frac{\left(w^{\left(2\right)}-w^{\left(1\right)}\right)x^{\prime }}{w^{\left(1\right)}d+\left(w^{\left(2\right)}-w^{\left(1\right)}\right)z^{\prime }}\\ \tan\beta ==\frac{\left(h^{\left(2\right)}-h^{\left(1\right)}\right)y^{\prime }}{h^{\left(1\right)}d+\left(h^{\left(2\right)}-h^{\left(1\right)}\right)z^{\prime }} \end{array}$$

introduce the *z*-dependence of UIM parameters.

### 2.2 Performance of UIM coupler

**Figure 2:**
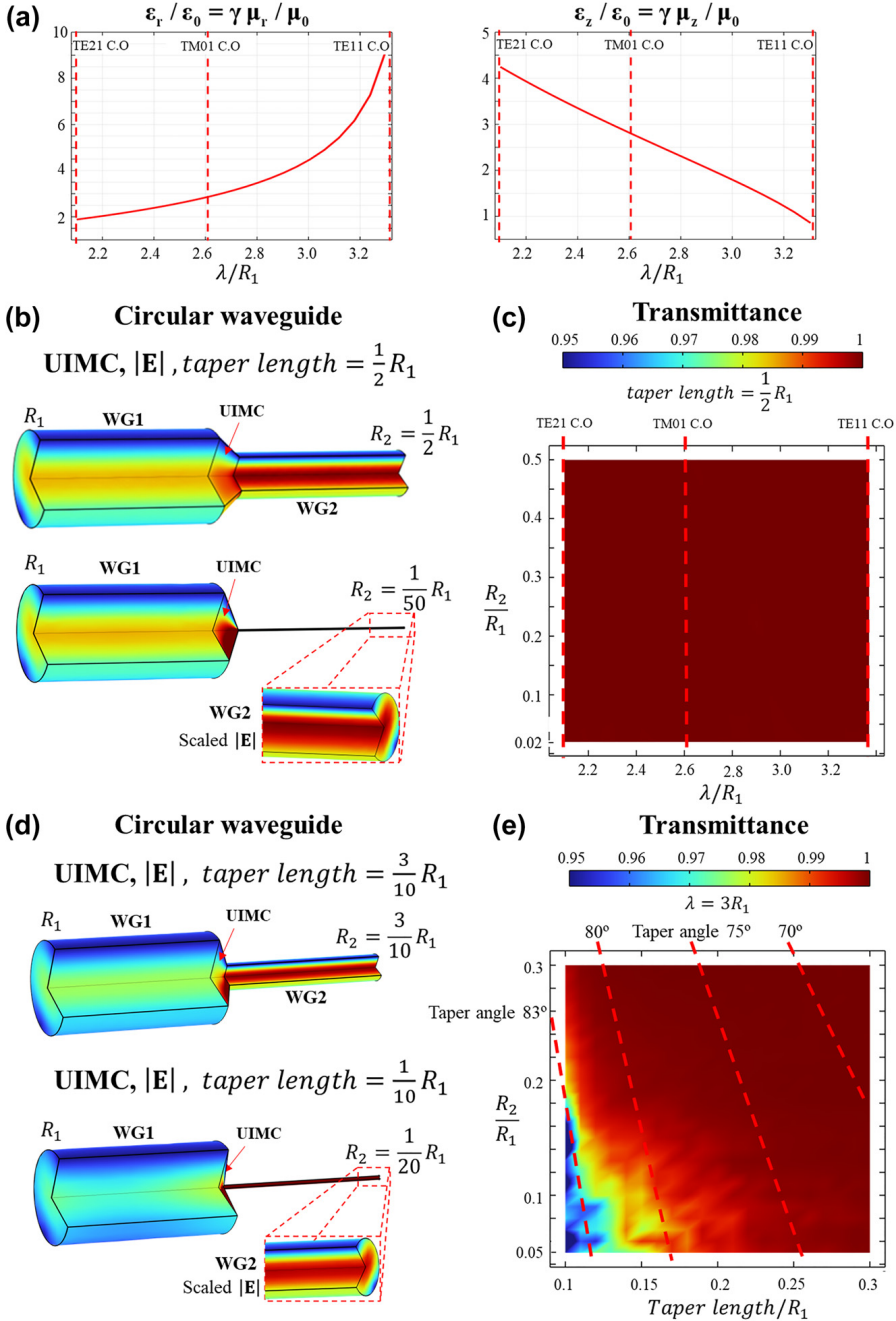
Performance of UIMC when coupling two PEC circular waveguides with same cut-off frequency. (a) The UIM parameter for $\varepsilon^{\prime \left(1\right)}=\varepsilon_{0}{,\varepsilon }^{\prime \left(2\right)}={4\varepsilon }_{0},\mu^{\prime \left(1\right)}=\mu^{\prime \left(2\right)}=\mu_{0}$ , where $\gamma =\mu_{0}\sqrt{Y^{\left(1\right)}Y^{\left(2\right)}}{/\varepsilon }_{0}\sqrt{Z^{\left(1\right)}Z^{\left(2\right)}}$ . Red dashed lines indicate the cut-off frequencies of the modes. (b and c) Examining the effect of varying *R*_2_ while keeping the UIMC taper length and *R*_1_ constant: (b) E-field images for the longest and shortest values of *R*_2_. (c) Resulting transmittance across a wide wavelength range and various *R*_2_ values. Red dashed lines indicate the cut-off frequencies of the modes. (d and e) Indicating the effects of varying both the taper length and *R*_2_ near the cut-off frequency of the dominant mode, TE11 mode: (d) E-field images for combinations of the longest and shortest values of *R*_2_ and taper length. (e) Corresponding transmittance results. Red dashed lines represent the taper angle.

**Figure 3:**
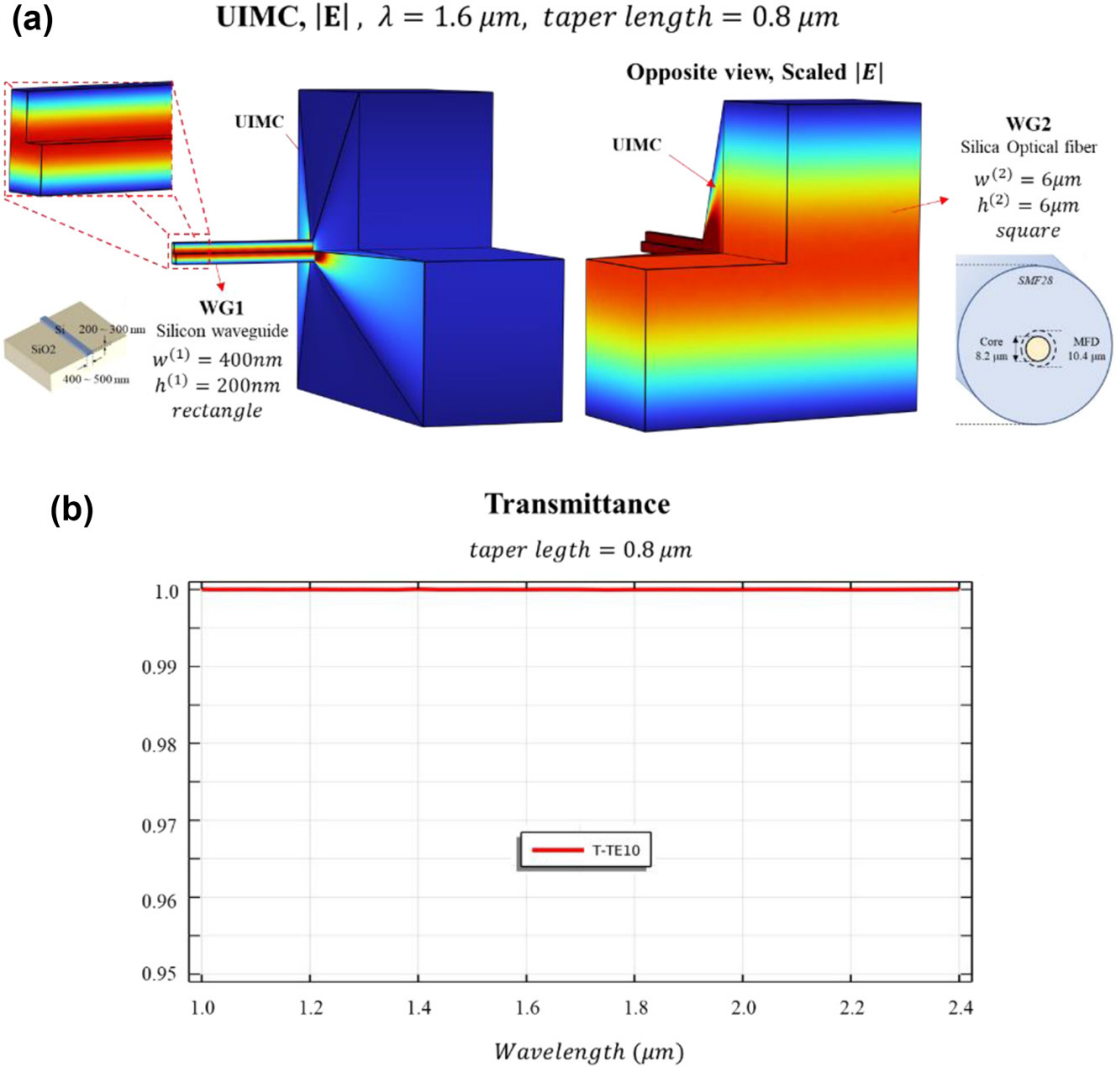
Performance of UIMC when coupling a square waveguide and a rectangular waveguide that emulate fiber-to-chip coupling. (a) Electric field images representing the coupling between WG1 (similar to a silicon waveguide chip) and WG2 (similar to optical fiber SMF-28), emulating fiber-to-chip coupling. (b) Corresponding transmittance over the NIR spectrum.

We employed COMSOL Multiphysics to simulate and validate the effectiveness of the Universal Impedance Matching Coupler (UIMC), a tapered coupler designed to eliminate reflections by addressing shape and impedance discrepancies. Figures 2 and 3 illustrate the performance of the UIMC for circular and rectangular waveguides, respectively. Within the circular waveguide featuring a PEC boundary, TM and TE modes are identified by the zeros of the Bessel function and its derivative, respectively (specific details have been omitted). To standardize the cross sections of WG1, with radius *R*_1_, and WG2, with radius *R*_2_, to a common reference cross section, radial direction scaling alone is adequate. This can be effectively accomplished by applying identical scaling factors to both the x and y dimensions (expressed as $a=b,w^{\left(j\right)}=h^{\left(j\right)}$ , $N^{\left(j\right)}=1$ in equations (3)–(6)). We assume uniaxial permittivity and permeability with *ɛ*_*x*_ = *ɛ*_*y*_ and *μ*_*x*_ = *μ*_*y*_. To derive the UIMC solution for the circular waveguide, we substitute $k_{x}^{2}+k_{y}^{2}$ by $k_{r}^{2}$ in equations (12) and (15) and then apply the transformation

(20) $$\begin{array}{c} x^{\prime }=R\left(z\right)x,\;y^{\prime }=R\left(z\right)y,\;z^{\prime }=z\\ R\left(z\right)=\frac{1}{ad}\left[R_{1}d+\left(R_{2}-R_{1}\right)z\right]\\ \tan\alpha ==\frac{\left(R_{2}-R_{1}\right)x^{\prime }}{\left[R_{1}d+\left(R_{2}-R_{1}\right)z^{\prime }\right],},\\ \tan\beta ==\frac{\left(R_{2}-R_{1}\right)y^{\prime }}{\left[R_{1}d+\left(R_{2}-R_{1}\right)z^{\prime }\right]}. \end{array}$$

In the simulation, WG1 maintained a constant $R_{1}=w^{\left(1\right)}=h^{\left(1\right)}$ while the radius $R_{2}=w^{\left(2\right)}=h^{\left(2\right)}$ of WG2 was altered for examination. WG1 was assumed to have air as the inner material, and the refractive index inside WG2 was set as *R*_1_/*R*_2_ to ensure that the cutoff frequencies of the modes within WG1 and WG2 were identical.

Figure 2 presents outcomes for different aspect ratios between WG1 and WG2, as well as varying taper lengths of the UIMC. In Figure 2(a) and (b), where the taper length is fixed at 1/2 *R*_1_, the performance is compared across a wide bandwidth while varying the aspect ratios of waveguide radii. Figure 2-(a) illustrates the magnitude of the E-field propagating through the UIMC. To provide a clearer visual representation, the interior regions of WG1, WG2, and UIMC were depicted by removing 1/4 of their structures. In Figure 2-(b), a surface plot showcases transmittance over different aspect ratios and wavelengths. It clearly demonstrates perfect transmission of the TE11 mode. The red dashed lines indicate the positions of the cutoff frequencies for the TE21, TM01, and TE11 modes. Calculations for the TM01 mode also indicate perfect transmission below the cutoff wavelength, indicating no coupling to other modes. These results affirm the consistency of perfect transmission regardless of the *R*_2_/*R*_1_ aspect ratio.

In Figure 2-(c) and (d), we examined the impact of taper length and aspect ratio on the transmission rate to observe the effect of the taper angle. The simulations were conducted by maintaining the frequency close to the cutoff frequency of the TE11 mode to exclusively excite this specific mode. Figure 2-(c) displays two contrasting scenarios with taper lengths of 3/10 and 1/10, along with aspect ratios of 3/10 and 1/20, respectively. Remarkably, even under extreme aspect ratios and short taper lengths, very high transmission rates were achieved. In Figure 2-(d), several tapering angles are represented by red dashed lines. It becomes evident that for taper angles approximately 75° or less, nearly 100 % transmission was observed. However, beyond this point, as the taper angle increased, the transmission rate declined, dropping to 95 % at an angle of 83°. This reduction in transmission rate beyond 75° is due to numerical discretization errors. In summary, our numerical analysis demonstrated an average broadband transmission rate of 99.9 % using the UIMC, even with an ultrashort tapering length. This suggests that the UIMC could potentially overcome the limitations of conventional fiber-to-chip couplers.

Figure 3 displays simulation outcomes intended to demonstrate UIMC’s potential in overcoming the earlier challenges associated with fiber-to-chip coupling. Unlike the circular waveguide with a similar cut-off frequency, the scenario involving the chip and fiber (WG1 and WG2) is more comprehensive. More specifically, WG1 represents a silicon waveguide within the simulation, which is depicted as a PEC rectangular waveguide with a width denoted as $w^{\left(1\right)}=400\;\text{nm}$ , utilizing silicon as the inner material. Conversely, WG2 models the SMF-28 optical fiber within the simulation, resembling a PEC square waveguide with both dimensions set at $w^{\left(2\right)}=h^{\left(2\right)}=6\;\mu \text{m}$ , and using silica as the inner material. A guided mode of the dielectric waveguide used in actual fiber to chip coupling, unlike the PEC waveguide, exhibits a spatial extent outside the waveguide in the form of evanescent waves. In the case of WG1, the shape of the guided mode inside the silicon waveguide is similar to the TE10 mode of the PEC rectangular waveguide, so it exactly imitates the size of the silicon waveguide. On the other hand, in the case of WG2, the mode shape of the single mode fiber (SMF) is different from the TE10 mode of the square waveguide, so only the size was imitated by the mode field diameter. Despite the actual shape of the SMF-28 optical fiber possessing a mode field diameter of approximately 10 μm, we constrain it to 6 μm due to computational limitations.

We achieved coupling between the two distinct waveguides, WG1 and WG2, utilizing UIMC with a 75-degree taper angle. This specific angle represents the maximum taper angle that enables nearly perfect transmission, as validated in previous findings. The UIMC’s length is 0.8 μm, shorter than the wavelength. Our simulation spanned a broad wavelength range within the central NIR band of optical communication. In Figure 3-(a), the E-field configuration validates the simulation’s accuracy for the TE10 mode. The interiors of WG1, WG2, and UIMC were plotted by a 1/4 cutout. In Figure 3-(b), a transmission rate graph displays almost 100 % transmission across the entire NIR spectrum, specifically illustrating the transmission rate for the TE10 mode. Although WG1 predominantly sustains the TE10 mode exclusively, WG2 exhibits dominant modes as both TE10 and TE01. Notably, around the wavelength of 1.6 μm, approximately 200 modes can be allowed within WG2. However, in WG2, it was confirmed that only TE10 mode was excited and that coupling to the other mode does not occur. Therefore, as evident in Figure 3-(a) and -(b), the successful occurrence of coupling exclusively within the same mode number demonstrates effective matching of mode shapes. Additionally, the near 100 % transmission rate indicates the successful matching of impedances.

## 3 Conclusions

We introduced the Universal Impedance Matching Coupler (UIMC), which addresses mode shape and impedance disparities analytically. UIMC facilitates the seamless transmission of electromagnetic waves, irrespective of the coupling conditions. The efficacy of UIMC was validated numerically using COMSOL Multiphysics. The UIMC parameter may seem quite complex, but in essence, it represents the UIM parameter undergoing a gradual spatial variation. This innovative coupler can be implemented using metamaterials to replicate the graded index characteristics of UIMC parameters. As a result, UIMC exhibits promising potential for efficient fiber-to-chip coupling. Conventional fiber-to-chip couplers typically suffer from limitations such as extended coupling lengths (with taper angles less than 25°), narrow bandwidths, and low coupling efficiencies (below 90 %). Until now, no solution has effectively addressed all these limitations simultaneously. However, our simulation outcomes highlight that UIMC successfully overcomes these constraints, achieving nearly flawless coupling.
